# Cost of three models of care for drug-resistant tuberculosis patients in Nigeria

**DOI:** 10.1186/s12879-018-3636-1

**Published:** 2019-01-10

**Authors:** Florence O. Bada, Evaezi Okpokoro, Nick Blok, Emmanuel Meribole, Saswata Dutt, Patrick Dakum, Alash’le Abimiku, Alice Zwerling, Sandra V. Kik

**Affiliations:** 1 International Research Center of Excellence, Institute of Human Virology Nigeria, 252 Herbert Macaulay Way, Central Business District, Abuja, Nigeria; 2 KNCV Tuberculosis Foundation, The Hague, The Netherlands; 3 Department of Health, Planning, Research and Statistics, Federal Ministry of Health, Abuja, Nigeria; 4 University of Maryland School of Medicine, Baltimore, MD USA; 5 School of Epidemiology and Public Health, University of Ottawa, Ottawa, Canada; 6 Academic Medical Center, Amsterdam Institute for Global Health and Development, Amsterdam, Netherlands

**Keywords:** Drug-resistant tuberculosis, Decentralized ambulatory care, Hospitalization, Cost

## Abstract

**Background:**

Nigeria accounts for a significant proportion of the global drug-resistant tuberculosis (DR-TB) burden, a large proportion of which goes untreated. Different models for managing DR-TB treatment with varying levels of hospitalization are in use across Nigeria, however costing evidence is required to guide the scale up of DR-TB care. We aimed to estimate and compare the costs of different DR-TB treatment and care models in Nigeria.

**Methods:**

We estimated the costs associated with three models of DR-TB treatment and care: Model (A) patients are hospitalized throughout the 8-month intensive phase, Model (B) patients are partially hospitalized during the intensive phase and Model (C) is entirely ambulatory. Costs of treatment, in-patient and outpatient care and diagnostic and monitoring tests were collected using a standardized data collection sheet from six sites through an ingredient’s approach and cost models were based on the Nigerian National Tuberculosis, Leprosy and Buruli Ulcer Guideline - Sixth Edition (2014) and Guideline for programmatic and clinical management of drug-resistant tuberculosis in Nigeria (2015).

**Results:**

Assuming adherence to the Nigerian DR-TB guidelines, the per patient cost of Model A was $18,528 USD, Model B $15,159 USD and Model C $9425 USD. Major drivers of cost included hospitalization (Models A and B) and costs of out-patient consultations and supervision (Model C).

**Conclusion:**

Utilizing a decentralized ambulatory model, is a more economically viable approach for the expansion of DR-TB care in Nigeria, given that patient beds for DR-TB treatment and care are limited and costs of hospitalized treatment are considerably more expensive than ambulatory models. Scale-up of less expensive ambulatory care models should be carefully considered in particular, when treatment efficacy is demonstrated to be similar across the different models to allow for patients not requiring hospitalization to be cared for in the least expensive way.

## Background

Nigeria has the second highest burden of tuberculosis (TB) in Africa and is among the 30 high drug-resistant tuberculosis (DR-TB) burden countries with an estimated 4700 patients with drug-resistant-TB (DR-TB) in 2015. Of these, only 1241 DR-TB patients were identified and 656 placed on treatment in Nigeria [1]. The treatment gap for DR-TB patients remains large and may even be increasing as case detection improves with increased usage of the GeneXpert® MTB/RIF technology that allows for the rapid diagnosis of rifampicin resistant TB (RR-TB). This rapidly evolving landscape is a clarion call to policymakers and practitioners to respond with improvement and scale up of treatment delivery for DR-TB.

Nigeria commenced programmatic management of DR-TB (PMDT) in 2010 following approval by the Green Light Committee [2] and started with hospitalization of patients at specialized treatment centers for the entire 8-month intensive phase followed by decentralized ambulatory care for the 12-month continuation phase. In 2013, community PMDT was initiated with patients receiving their entire course of treatment via ambulatory care. This was in line with a wide-spread move towards reducing lengthy hospitalizations for DR-TB patients and promoting ambulatory care models where the patient can remain at home in the community during part of their treatment course. A systematic review comparing hospitalization with community based DR-TB care during the intensive phase suggested that both DR-TB models of care provide comparable treatment success rates, lending further support to the 2011 World Health Organization (WHO) recommendation as part of updated DR-TB guidelines to include ambulatory models for DR-TB treatment [3, 4].

Treating DR-TB is extremely costly [5], yet data remains limited around costs associated with various models of DR-TB care [6–9]. More accurate costs for treating DR-TB using various models are required for rational planning and allocation of resources, to determine optimal models of care and to prioritize competing health care issues.

Huge investments are needed to scale-up DR-TB care in Nigeria; either towards community systems’ strengthening for utilization of ambulatory DR-TB treatment models or to scale-up infrastructure for hospital-based DR-TB models of care. The amount of resources required to achieve this are not readily available to the NTBLCP given an unfunded budget for TB control of 55% in Nigeria in 2015 [1]. Thus, it is imperative that evidence be provided to guide investments towards the scale-up of the DR-TB program in Nigeria, while assuring the provision of high quality care for patients.

We performed a comprehensive cost analysis of the three different models of DR-TB treatment and care in Nigeria employed in the period from June 2013 through December 2014.

## Methods

### Setting

Nigeria has a per capita income of $2178 USD [10] and an estimated DR-TB prevalence of 4.3% among new and 25% among retreatment TB cases [1]. Management of identified DR-TB cases is based on a standardized WHO approved treatment regimen of 20 months consisting of an 8-month intensive phase and a 12-month continuation phase. Patients are placed on pyrazinamide and four second-line anti-TB drugs namely levofloxacin, kanamycin (replaced by capreomycin when indicated), prothionamide and cycloserine. All five drugs are used for the 8-month intensive phase at the end of which kanamycin (or capreomycin) is discontinued for the remaining 12-month continuation phase.

Three models of DR-TB care were utilized in Nigeria between June 2013 and December 2014 and differed only in their 8-month intensive phase. Patients treated under Model A, were hospitalized for the complete duration of the intensive phase; patients in Model B were hospitalized for a duration of 5 months in the intensive phase while patients treated under Model C received the complete intensive phase treatment as ambulatory care in the community. The continuation phase was identical in each of the three models with directly observed treatment (DOT) provided daily in the community by a treatment supporter supplemented by visits to the DOT center every 2 weeks for drug pick-ups. Patients were also monitored via bi-weekly home visits by the specific DOT officer overseeing their care, monthly home visits by the TB and Leprosy Supervisor (TBLS) overseeing the local government in which they were receiving treatment, monthly home visits by the state DR-TB focal person and quarterly home visits by the state consilium which is a multi-disciplinary DR-TB team consisting of a state TB control officer, a state quality assurance officer who is usually also the laboratory focal person, the state logistics officer who also doubles as the state pharmacist, an ear, nose and throat (ENT) surgeon/audiometrist, the state DR-TB focal person, a nurse from the treatment center or DOT center, a chest physician, a psychiatrist, a monitoring and evaluation focal person and a social worker.

Patients were assigned to one of the three models by health care professionals based on the patient’s medical history, the availability of the model in their geographical region and the patient’s health status at the time of commencing treatment taking into cognizance the patient’s preference.

### Costing

We performed a cost analysis to better understand the costs and important drivers of cost across the different models of DR-TB management. The cost analysis was performed from the perspective of the Nigerian NTP (health sector perspective) and includes all DR-TB related management costs such as diagnostic and monitoring tests, hospitalization, clinic visits, home visits, treatment supervision and drugs. We assumed strict adherence to the Nigerian NTBLCP Guideline for programmatic and clinical management of drug-resistant tuberculosis in Nigeria (2015) and no difference in the effectiveness of the treatment in the analysis (i.e. a cost minimization). The time horizon for the analysis was 20 months which is the length of a full course of DR-TB treatment. All costs were collected in NGN for the year 2014. Final costs are expressed in 2014 USD using the average exchange rate in 2014 of 1 USD = 158 NGN [11]. Costs were not inflated to 2017 dollars due to the considerable change in exchange rate for the Naira between 2014 and 2017.

### Unit costs

#### Inpatient, outpatient and clinic costs

Cost data were obtained from six health facilities; three treatment centers providing hospitalization as part of care and three DOT centers providing ambulatory DR-TB care. Costs were built using an ingredients approach, also called bottom-up approach whereby each resource or cost item required was identified and valued in order to calculate the unit cost. Costs were collected using a standardized data collection sheet at each site. To estimate the total cost of hospitalization, we averaged the costs from three treatment centers providing hospitalization as part of care. Likewise, to estimate the total cost of out-patient services, we averaged the costs from three DOT centers providing ambulatory DR-TB care.

Treatment center 1 is a secondary faith-based hospital in Nigeria’s South-West Geopolitical zone, Treatment center 2 is a tertiary treatment and training center located in Nigeria’s North West Geo-political zone and Treatment center 3 is a public secondary treatment center located in the South West Geo-political zone. Capital costs collected included equipment, vehicles and furniture and were annualized using a discounted rate of 3%, and the assumption of 10 years of useful life. Information on staff salaries and grade levels were obtained from administrative documents and staff interviews. Cost for personnel and other recurrent costs, including utilities and maintenance costs, ancillary costs, including catering and laundry services, and the purchase, maintenance and operation of infrastructure and equipment were all collected from hospital expenditure and financial reports. Shared costs including salaries, furniture, supervision, transportation and vehicles were estimated through observation and interviews with health staff. In addition, costs for specific items were obtained from asset registers and procurement lists from different institutes supporting DR-TB care in Nigeria including Institute of Human Virology, Nigeria (IHVN), Damian Foundation and KNCV Tuberculosis Foundation. Total inpatient and outpatient costs were divided by the total number of inpatient days and outpatient visits to calculate the cost per inpatient day and outpatient visit, respectively.

Costs made in the community were collected from three DOT centers that provided ambulatory DR-TB care – DOT Center 1 was located in the South-South Geo-political zone, DOT Center 2 in the South-West Geopolitical zone and DOT Center 3 in the North-West Geo-political zone. Costs were obtained from hospital expenditure reports and interviews with staff. Outpatient services were assumed to use a proportion of hospital overhead costs [12] .

The time spent by each staff member of the state consilium on different types of visits to or from DR-TB patients or for related meetings, as well as the stipends given to these state staff for transportation and communication as they oversee the management of DR-TB patients, were obtained via staff interviews. Funds spent to convene meetings and for the transportation of staff for home visits were obtained from financial and meeting reports.

#### Diagnostic and monitoring test costs

Diagnostic and monitoring tests were performed as recommended in the NTBLCP management and control guidelines for the programmatic and clinical management of DR-TB. The costs of diagnostic and monitoring TB tests such as smear microscopy, Xpert, chest X-ray, culture (liquid and solid) and drug susceptibility testing (DST) were determined using an ingredients approach. This included costs of reagents and consumables, equipment, power supply and salaries of the laboratory personnel obtained from two laboratories; Laboratory 1 (Lab 1) is the National Reference Laboratory located in the North-West Geopolitical zone and Laboratory 2 (Lab 2) is a Private Laboratory stationed in the North Central Geo-political zone.

Salary costs were estimated in two ways; laboratory personnel were asked to estimate the time needed to perform a single test (or a batch of tests if appropriate) (bottom-up) and laboratory personnel performing each test were asked to estimate what proportion of their time they spent on each test (top-down) and both estimates were averaged. The cost of audiometric testing was obtained from the financial reports of three treatment centers. For chemistry and hematology tests, the actual costs were obtained from five commercial laboratories and averaged.

#### Drug costs

The drug costs were based on the Global Drug Facility price list which is the sole source for all the drugs used for DR-TB management in Nigeria.

#### Frequencies

Frequencies of visits, drugs used, tests, visits and hospital days in each of the 3 models were derived from the Nigerian national guideline for programmatic and clinical management of DR-TB in Nigeria (2015) (See appendix). For our analysis, we assumed strict adherence to the national guidelines even though we recognize that under routine circumstances this might not always be done.

Total health care costs for each model were calculated using the DR-TB cost-effectiveness analysis (CEA) -Tool (Version 1) which was developed by Management Sciences for Health (MSH) through the USAID-funded TB CARE 1 project and has been used and validated to determine health care costs for different DR-TB treatment options in Indonesia. [13]

The average costs for the diagnosis, treatment and care during the intensive and the continuation phases of treatment are described for each of the treatment models.

### Ethical issues

The study spanned several states in the Federal Republic of Nigeria and entailed direct observation of health care workers (HCWs) as they cared for DR-TB patients as well as interviewing HCWs to obtain the required information to calculate the health service costs of providing care to DR-TB patients. Written informed consent was obtained from HCWs prior to interviewing them. The protocol was approved by the National Health Research Ethics Committee, Nigeria (NHREC).

## Results

### Inpatient costs

The costs constituting one bed day in a hospital for DR-TB patients are provided in Table 1. Costs for all components were consistently lower in Treatment Center 1 ($22.91 USD) compared with Treatment Center 2 ($87.67 USD) and 3 ($49.0 USD), with Treatment Center 3 over two times as costly as Treatment Center 1 and Treatment Center 2 three times more costly for one hospital bed day than Treatment Center 1. Salaries were an important driver of cost across all hospitals (27.7, 25.8 and 42.2%). Administrative costs were an important driver of cost for hospitals 2 and 3 (30.9 and 34.4% respectively) while catering for patient’s food (33%) was the highest cost component for Treatment Center 1.

**Table 1:** Costs for one bed day in a hospital for a DR-TB patient in USD

Cost Component	Treatment Center 1 (%)	Treatment Center 2 (%)	Treatment Center 3 (%)	Average (%)
Administration	3.74 (16.3)	27.12 (30.9)	16.88 (34.4)	15.91 (30)
Transportation	1.83 (8.0)	11.97 (13.7)	-^a^	4.60 (9)
Laundry	0.25 (1.1)	3.27 (3.7)	2.84 (5.8)	2.12 (4)
Radiology	0.77 (2.1)	7.11 (6.1)	-^b^	2.62 (5)
Maintenance cleaning and security	2.16 (9.4)	5.25 (6.0)	1.82 (3.7)	3.08 (6)
Catering	7.57 (33)	9.46 (10.8)	6.31 (12.9)	7.78 (15)
Other supplies	0.24 (1.0)	0.89 (1.0)	0.48 (1.0)	0.54 (1)
Salaries	6.35 (27.7)	22.61 (25.8)	20.67 (42.2)	16.54 (31)
Total costs one bed day	22.91 (100)	87.67 (100)	49.00 (100)	53.19 (100)

^a^This treatment center had no official cars and no drivers so no costs could be attributed to transportation

^b^This treatment center had no functioning radiological equipment during the period over which costing information was obtained; patients were referred out for chest X-rays when needed

### Outpatient costs

Costs for the different kinds of outpatient visits, conducted by the patient or the treatment supervisor(s) or teams varied slightly across the three DOT centers evaluated (Table 2). Salary costs were the main driver across all types of outpatient visits. Among the home visits, quarterly state team home visits were consistently the most costly outpatient visit ranging from $132.89 USD to $248.90 USD per visit with salary costs and costs for staff travel as main drivers of cost across all three States. Regular DOT provided at DOT centers during the intensive phase was consistently the least expensive outpatient visit ranging from $4.19 to $4.76 USD per visit across the three sites evaluated.

**Table 2:** Costs for outpatient consultations, home visits and treatment supervision in USD

Cost Component	DOT 1	DOT 2	DOT 3	Average (%)
Monthly clinic visits				
Salary costs	16.49	11.33	3.25	10.36 (64)
Overhead	3.1	1.95	0.63	1.89 (12)
Transport	3.16	3.16	3.16	3.16 (19)
Other costs^	1.76	0.16	0.49	0.80 (5)
Total	24.51	16.60	7.53	16.21 (100)
DOT at DOT Center – Intensive phase				
Salary costs	0.88	0.58	1.17	0.88 (20)
Overhead	0.15	0.51	0.23	0.30 (7)
Transport	3.16	3.16	3.16	3.16 (71)
Other costs^	0	0.15	0.2	0.12 (3)
Total	4.19	4.40	4.76	4.45 (100)
DOT at DOT Center – Continuation phase				
Salary costs	0.88	0.58	1.17	0.88 (20)
Overhead	0.15	0.51	0.23	0.33 (7)
Transport	3.16	3.16	3.16	3.16 (71)
Other costs^a^	0	0.15	0.2	0.12 (3)
Total	4.19	4.40	4.76	4.45 (100)
Home visit by DOT Officer – Intensive Phase				
Salary costs (visit)	4.03		2.34	3.19 (28)
Salary costs (travel time)	4.03		3.50	3.77 (34)
Transport	3.16		3.16	3.16 (28)
Other costs^a^	1.60		0.59	1.10 (10)
Total	12.82	NA+	9.59	11.21 (100)
Home visit by DOT Officer – Continuation Phase				
Salary costs (visit)	4.03		2.34	3.19 (29)
Salary costs (travel time)	4.03		3.50	3.77 (34)
Transport	3.16		3.16	3.16 (28)
Other costs^a^	1.60		0.40	1.00 (9)
Total	12.85	NA^b^	9.40	11.13 (100)
Home visit by DRTB Focal Person				
Salary costs (visit)	6.28			6.28 (19)
Salary costs (travel time)	20.94			20.94 (65)
Transport	3.16			3.16 (10)
Other costs^a^	1.93			1.93 (6)
Total	32.32	NA+	NA+	32.32 (100)
Home visit by TBL Supervisor				
Salary costs (visit)	2.01	2.01	2.09	2.04 (20)
Salary costs (travel time)	4.03	4.03	6.28	4.78 (46)
Transport	3.16	3.16	3.16	3.16 (31)
Other costs^a^	1.03	0.09	0	0.37 (4)
Total	10.23	9.29	11.53	10.35 (100)
Quarterly State Team Home visit				
Salary costs (visit)	163.4	48.61	214.04	142.02 (76)
Salary costs (travel time)	0^c^	81.03	26.76	35.93 (19)
Transport	3.16	3.16	3.16	3.16 (2)
Other costs^a^	10.04	0.09	4.94	5.02 (3)
Total	176.60	132.89	248.90	186.13 (100)
Consilium Meeting				
Salary	58.48	26.81	49.51	44.93 (75)
Allowances	0	9.04	20.1	9.71 (16)
Catering	0	5.42	0	1.81 (3)
Other costs^a^	10.13	0	0	3.38 (6)
Total	68.61	41.27	69.61	59.83 (100)

*DRTB* drug resistant tuberculosis, *NA* not available, *TBL* tuberculosis and leprosy

^a^Other costs include the cost of supplies such as gloves, face masks, N-95 respirators, hand sanitizer, syringes and needles and spirit swabs

^b^No available data for DOT Officer and DR-TB Focal Person during the data collection exercise in their states

^c^It was not possible to separate travel time from visit time; therefore both are captured under Salary costs (visit)

### Diagnostic and monitoring test costs

Costs of TB tests were consistently higher in Lab 2 as compared to Lab 1 (Table 3). As expected [14], top down estimates for costing the contribution of staff salaries were usually higher compared with bottom up estimations. The cost for liquid culture ($97.20 USD on average) was about three times as expensive as the cost for solid culture ($39.43 USD on average). First line DST performed on liquid culture were the most expensive TB test cost ($130.44 USD on average). DST performed on solid culture, testing resistance against a panel of nine first and second line drugs were estimated to cost $68.23 on average, but these costs could only be estimated in one lab since the other lab did not perform second line DST. An average of top down and bottom up costs were used in the analysis. For smear microscopy the cost of Ziehl-Neelsen and fluorescence were averaged.

**Table 3:** Costs of tuberculosis tests in USD

	Lab 1		Lab 2		Average (%)
	Salaries bottom-up	Salaries top-down	Salaries bottom-up	Salaries top-down	
Cost of 1 AFB smear microscopy Ziehl-Neelsen					
Reagents	0.33	0.33	0.43	0.43	0.38 (6)
Supplies	1.36	1.36	4.90	4.90	3.13 (49)
Labor	0.91	1.74	1.55	1.23	1.36 (21)
Equipment	0.70	0.70	0.99	0.99	0.85 (13)
Overhead	0.36	0.36	0.87	0.87	0.62 (10)
Total	3.67	4.50	8.73	8.41	6.33 (100)
Cost of 1 AFB Smear microscopy fluorescence					
Reagents	0.30	0.30	0.30	0.30	0.30 (5)
Supplies	1.41	1.41	4.90	4.90	3.16 (20)
Labor	1.00	1.74	1.66	1.23	1.41 (22)
Equipment	0.72	0.72	0.99	0.99	0.86 (13)
Overhead	0.38	0.38	0.86	0.86	0.62 (10)
Total	3.80	4.55	8.72	8.28	6.34 (100)
Cost of solid culture^a^					
Reagents	12.04	12.04	13.73	13.73	12.89 (33)
Supplies	14.40	14.40	5.07	5.07	9.74 (25)
Labor	0.95	5.31	4.84	14.67	6.44 (16)
Equipment	6.40	6.40	8.57	8.57	7.49 (19)
Overhead	3.72	3.72	2.03	2.03	2.88 (7)
Total	37.51	41.87	34.24	44.08	39.43 (100)
Cost of liquid culture					
Reagents	12.00	12.00	14.84	14.84	13.42 (14)
Supplies	27.65	27.65	20.21	20.21	23.93 (25)
Labor	1.02	6.48	3.64	48.48	14.91 (15)
Equipment	22.35	22.35	52.25	52.25	37.30 (38)
Overhead	6.93	6.93	8.37	8.37	7.65 (8)
Total	69.94	75.41	99.31	144.15	97.20 (100)
Cost of first line DST (liquid culture)					
Reagents	9.59	9.59	9.50	9.50	9.55 (7)
Supplies	43.42	43.42	61.57	61.57	52.50 (40)
Labor	0.88	20.28	3.02	73.90	24.52 (19)
Equipment	16.43	16.43	50.88	50.88	33.66 (26)
Overhead	7.73	7.73	12.70	12.70	10.22 (8)
Total	78.05	97.45	137.68	208.56	130.44 (100)
Cost for first and second line DST (solid culture)					
Reagents	3.44	3.44			3.44 (5)
Supplies	41.52	41.52			41.52 (61)
Labor	1.42	28.39			14.90 (22)
Equipment	2.94	2.94			2.94 (4)
Overhead	5.43	5.43			5.43 (8)
Total	54.74	81.71	ND	ND	68.23 (100)
Cost of Xpert MTB/RIF test					
Reagents	9.98	9.98	9.98	9.98	9.98 (41)
Supplies	1.28	1.28	8.93	8.93	5.11 (21)
Labor	0.57	11.30	0.56	11.23	5.92 (24)
Equipment	2.28	2.28	3.49	3.49	2.89 (12)
Overhead	1.55	0.01	1.43	0.01	0.75 (3)
Total	15.67	24.85	24.38	33.64	24.64 (100)

List of abbreviations: *AFB* Acid-fast bacilli, *DST* drug susceptibility testing, *MTB Mycobacterium tuberculosis*, *ND* not done in this lab, *RIF* rifampicin, *TB* tuberculosis

^a^solid culture based on Lowenstein Jensen media

Costs for other monitoring tests and the costs for individual TB medications are shown in Table 4.

**Table 4:** Costs of ancillary monitoring tests and DR-TB medications in USD

	Cost per Unit
Ancillary monitoring tests	
Audiometry	31.65
Kidney function tests (E, U, Cr)	21.04
Thyroid Function Test	62.18
Urinalysis	2.69
Liver Function Test	22.26
Fasting Blood Sugar	4.85
Chest X-Ray	10.92
HIV Test	6.33
Pregnancy test	2.85
DR-TB medications	
Capreomycin 1 g (per vial)	4.7
Amikacin 500 mg (per vial)	0.62
Cycloserine 250 mg (per capsule)	0.29
Ethambutol 400 mg (per tab)	0.03
Prothionamide 250 mg (per tab)	0.07
Kanamycin 1 g vial (per vial)	0.24
Levofloxacin 250 mg (per caplet)	0.03
Pyrazinamide 400 mg (per tab)	0.02
Pyrazinamide 500 mg (per tab)	0.03
Pyridoxin 10 mg (per tab)	0.03

*Cr* creatinine, *DR-TB* drug resistant tuberculosis, *DST* drug susceptibility testing, *HIV* human immunodeficiency virus, *E* electrolytes, *U* urea

The cost of audiometric testing was obtained from the financial reports of three treatment centers. For C-Xrays, chemistry and hematology tests, the actual costs were obtained from five commercial laboratories and averaged. The drug costs were based on the Global Drug Facility price list

The number of diagnostic and monitoring tests and drugs, as prescribed in the Nigerian NTBLCP DR-TB guideline, were identical for the three different models of care (Table 5). However, the models differed in the number of inpatient hospitalization days (243 for Model A, 152 for Model B and none for Model C) and type and frequency of the treatment monitoring visits during the intensive and continuation phases.

**Table 5:** Frequencies of laboratory tests and patient monitoring visits per patient and model according to the Nigerian DR-TB guidelines

		Model A		Model B		Model C	
	Unit	Frequency in intensive phase	Frequency in continuation phase	Frequency in intensive phase	Frequency in continuation phase	Frequency in intensive phase	Frequency in continuation phase
I. DR-TB Diagnostic tests							
GeneXpert	Test	1	–	1	–	1	–
Sputum smear	Smear	1	–	1	–	1	–
Culture – Liquid	Test	1	–	1	–	1	–
1st and 2nd line DST - Solid culture	Test	1	–	1	–	1	–
II. Baseline tests & routine laboratory tests							
X-ray	Test	2	2	2	2	2	2
ENT consultation	Consult	1	–	1	–	1	–
Audiometry test	Test	9	–	9	–	9	–
E, U, Cr	Test	8	–	8	–	8	–
Thyroid function test	Test	2	2	2	2	2	2
LFT	Test	3	4	3	4	3	4
HIV Test	Test	1	–	1	–	1	–
Pregnancy test	Test	0.5	–	0.5	–	0.5	–
III. Drugs							
Pyrazinamide 400 mg	Tablet	972	1488	972	1488	972	1488
Kanamycin 1 g vial	Vial	243	–	243	–	243	–
Levofloxacin 250 mg	Caplet	729	1116	729	1116	729	1116
Prothionamide 250 mg	Tablet	729	1116	729	1116	729	1116
Cycloserine 250 mg	Capsule	729	1116	729	1116	729	1116
Pyridoxine 10 mg	Tablet	729	1116	729	1116	729	1116
IV. Inpatient stay							
Inpatient hospitalization days	Bed day	243	–	152	–	–	–
V. Outpatient consultations and supervision							
Consultation at treatment center /Monthly clinic visit	Visit	–	12	3	12	8	12
Visits to collect medication at DOT center/ DOT at DOT center	Visit	–	24	6	24	16	24
Home visit - by DOT officer	Home visit	–	24	91	24	243	24
Home visit by DR TB focal person	Home visit	–	12	3	12	8	12
Home visit by TBL supervisor	Home visit	–	12	3	12	8	12
Quarterly state team meeting	Meeting	–	4	1	4	3	4
Quarterly state team home visit	Home visit	–	4	2	4	2	4
VI. Follow-up DR-TB testing							
Sputum smear	Smear	8	12	8	12	8	12
Culture - Liquid	Test	8	6	8	6	8	6
1st and 2nd line DST - Solid culture	Test	0.2^	0	0.2^	0	0.2^a^	0

List of abbreviations: *Cr* creatinine, *DOT* directly observed treatment, *DR* drug resistant, *DST* drug susceptibility testing, *E* electrolytes, *ENT* ear nose throat, *TB* tuberculosis, *TBL* tuberculosis and leprosy, *U* urea, *LFT* liver function test,

^a^A conservative estimate of DR-TB patients who are still culture positive after four months of DR-TB treatment and thus require repeat second-line DST

**Fig. 1:**
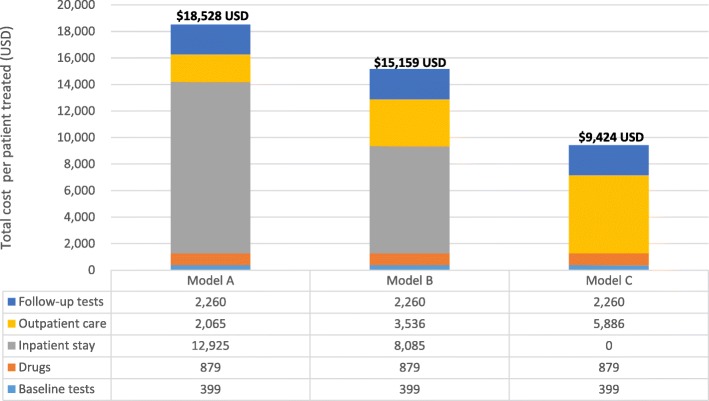
Total Costs for three models of DR-TB treatment care in Nigeria

We estimated, that the total cost to provide the diagnostic and treatment care as outlined in the Nigerian DR-TB guidelines, would be $18,528 USD per patient for Model A, $15,159 USD per patient for Model B and $9425 USD per patient for Model C (Fig. 1). For all models, the cost for patient care (either inpatient or outpatient) accounted for more than half of the total health service costs. Even though the frequency of home visits by the DOT officer or DR-TB focal person were substantially higher in the intensive phase for Model C, the total costs for home visits and out-patient visits ($5806 USD) were still much lower than those for the total or partial inpatient hospitalization in the intensive phase in Models A ($12,925 USD) and B ($8085 USD).

## Discussion

We performed this cost analysis to calculate the total costs associated with the management of DR-TB in Nigeria from the health sector perspective and the costs of individual components of DR-TB care including the costs for hospitalization and outpatient costs, costs of visits to the DOT center, home visits, treatment supervision, drug costs and costs of diagnostic and monitoring tests. To our knowledge, this is the first study that has quantified the costs of treating DR-TB in Nigeria utilizing different models of care according to the 2015 Nigerian DR-TB guidelines. Results from this study will facilitate rational planning and resource allocation as Nigeria scales up DR-TB care including ambulatory models of treatment delivery and the introduction of shorter DR-TB Treatment regimen, new TB drugs such as Delamanid and Bedaquiline as well as repurposed drugs like linezolid and clofazimine.

Our main findings showed that Model A with hospitalization for the entire 8-month intensive phase cost 18528USD (equivalent to 2,927,464 NGN in 2014) per treated patient, two times the cost as estimated for Model C (9425USD, equivalent to 1,489,080 NGN) where patients are managed through decentralized ambulatory care. The cost for Model B with hospitalization for 5 months during the intensive phase was 15159USD (equivalent to 2,395,070 NGN). Managing patients without hospitalization reduces the cost of DR-TB care by approximately 49% in the Nigerian setting. This is in line with findings from similar studies [15, 16], which showed that models with hospitalization were more costly compared to partial or fully ambulatory treatment models.

Hospitalization was responsible for over 70% of the cost of Model A while out-patient consultations and supervision and laboratory diagnostic and follow-up testing contributed 9 and 12% respectively. However, the level of treatment facility utilized for treatment (secondary or tertiary) significantly affected the cost of hospitalization with hospitalization in a tertiary hospital being more than twice the cost of hospitalization in a secondary public hospital and three times the cost of hospitalization in a secondary faith-based hospital according to our findings. This may be explained by the fact that treatment center 1 was a faith-based hospital with minimal support from the government and thus has less funds available for salaries and overhead costs while treatment center 2 which is supported by the Federal Government of Nigeria and treatment center 3 by a State Government are well funded with salaries and administrative costs associated with these two hospitals notably higher. Transportation costs in treatment center 2 accounted for 13.7% of the total hospitalization costs due to many vehicles owned and used by the tertiary hospital as well as the costs of fueling and maintaining the vehicles.

For Model C, outpatient consultations and supervision were responsible for over 62% of the cost, while laboratory diagnostic and monitoring tests accounted for 28%. These cost drivers are similar to those found in other studies [6, 16] with the exception of drugs which only contributed 5–9% across the three models in this study. Costs for the monthly clinic visits varied the most across the different states, mainly due to the different number and cadre of staff participating in these visits and the time spent with the patient during these visits. Quarterly state team home visits were consistently the most costly outpatient visit, ranging from $133USD to $249USD per visit with salary costs and costs for staff travel as main drivers across all three states and greatly influenced by the time spent journeying to the patients’ residences. Regular DOT provided at DOT centers during the intensive phase was consistently the least expensive outpatient visit ranging from $4.19 to $4.76 USD per visit across the three sites evaluated and should continue to be promoted as a feasible way to ensure optimal patient monitoring. However, we did not take the patient costs associated with these outpatient visits into account. Home visits by HCW though slightly more expensive can continue to be interspersed with patient visits to the DOT center and have the added advantage of providing a means for health care workers to ensure conditions in the patient’s home minimize the risk of TB transmission to family members and neighbors. The significant contribution of out-patient consultation and supervision to ambulatory models, emphasize findings from other studies that when shifting from hospital-based care to ambulatory systems, provision needs to be made for alternate health system support [17].

While outpatient consultations were more expensive for Models B and C with ambulatory care, these increased costs were still dwarfed by costs associated with hospitalization in Model A. The use of decentralized models have the added benefit of earlier treatment initiation and preserving the patient’s social support structure and are thought to have comparable success rates to models with some degree of hospitalization [3].

Salary costs are significant drivers of cost in all components of DR-TB care therefore, particularly among outpatient consultation and monitoring, optimizing human resources in these activities is critical in future scale-up activities. Exploring task shifting of patient supervision may increase coverage of patient supervision and yield considerable cost savings.

There are a number of limitations to our study. We were not able to include building costs which are a substantial cost because land for the construction of government hospitals is not purchased but appropriated for the purpose and there are no standard rates for renting per square meter which could have served as a proxy for building costs. We were also not able to include costs for ancillary medications given to patients and general supplies utilized by patients while hospitalized because although the total costs spent on ancillary medication and hospital supplies were obtained for each treatment center, insufficient utilization information was available to allocate proportionate costs to DR-TB patients hospitalized at the treatment centers in these health facilities.

Frequency of diagnostic and monitoring tests and outpatient consultations were all based on the 2015 Nigerian DR-TB guidelines, while in reality individual patients may undergo more or fewer tests and visits depending on their situation. We solely described the cost for the complete treatment of a patient with DR-TB when cared for under one of three models of DR-TB care available in Nigeria, without comparing the effectiveness of the different models. During the study period, DR-TB patients were allocated to a model based on availability and the preference of the patient and clinician. Severely ill patients might have been assigned more often to model A based on their clinical condition. Additional studies are needed to build evidence around the most cost-effective model for different patients taking their disease characteristics and clinical condition into account. The cost-effectiveness of each of the models will be published in a later study which will take into account actual frequencies of visits and resources used by individual patients.

The use of cost data from treatment centers housed in three different types of hospitals might not reflect the whole of Nigeria. However, hospitals were selected from different geographical zones to achieve a representative picture of DR-TB costs and ranged from a National Tertiary hospital to a small district hospital.

The strengths of the study include the evaluation of three treatment models within the same geopolitical setting and the collection of detailed data used to build unit costs for Nigeria instead of using previously published estimates or projections from other settings.

## Conclusion

This costing study demonstrates that DR-TB treatment models employing ambulatory care are considerably less costly compared to approaches that utilize hospitalization. Scale-up of less expensive ambulatory care models should be carefully considered in particular, when treatment efficacy is demonstrated to be similar across the different models to allow for patients not requiring hospitalization to be cared for in the least expensive way in Nigeria to address the large treatment gap in treating DR-TB patients.

## Abbreviations

AFB: Acid-fast bacilli
CEA: Cost-effectiveness analysis
Cr: Creatinine
DOT: Directly observed treatment
DR-TB: Drug-resistant tuberculosis
DST: Drug susceptibility testing
E: Electrolytes
ENT: Ear, nose and throat
HCW: Health care worker
HIV: Human immunodeficiency virus
LFT: Liver function test
MSH: Management sciences for health
MTB: Mycobacterium tuberculosis
NA: not available
ND: not done
NGN: Nigerian naira
NTBLCP: National tuberculosis and leprosy control programme
NTP: National tuberculosis programs
PMDT: Programmatic management of drug-resistant TB
RIF: Rifampicin
TB: Tuberculosis
TBL: Tuberculosis and leprosy
TBLS: Tuberculosis and leprosy supervisor
U: Urea
USD: United States dollars
WHO: World Health Organization
